# Methylation index of the DLK1 and MKRN3 genes in precocious puberty

**DOI:** 10.18699/vjgb-25-47

**Published:** 2025-06

**Authors:** E.A. Sazhenova, O.Yu. Vasilyeva, D.A. Fedotov, M.B. Kankanam Pathiranage, A.D. Lobanov, A.Yu. Sambyalova, E.E. Khramova, L.V. Rychkova, S.A. Vasilyev, I.N. Lebedev

**Affiliations:** Scientific Research Institute of Medical Genetics, Tomsk National Research Medical Center of the Russian Academy of Sciences, Tomsk, Russia; Scientific Research Institute of Medical Genetics, Tomsk National Research Medical Center of the Russian Academy of Sciences, Tomsk, Russia; Scientific Research Institute of Medical Genetics, Tomsk National Research Medical Center of the Russian Academy of Sciences, Tomsk, Russia; Tomsk State University, Tomsk, Russia; Siberian State Medical University, Tomsk, Russia; Scientific Center for Family Health and Human Reproduction Problems, Irkutsk, Russia; Scientific Center for Family Health and Human Reproduction Problems, Irkutsk, Russia; Scientific Center for Family Health and Human Reproduction Problems, Irkutsk, Russia; Scientific Research Institute of Medical Genetics, Tomsk National Research Medical Center of the Russian Academy of Sciences, Tomsk, Russia Tomsk State University, Tomsk, Russia; Scientific Research Institute of Medical Genetics, Tomsk National Research Medical Center of the Russian Academy of Sciences, Tomsk, Russia Siberian State Medical University, Tomsk, Russia

**Keywords:** precocious puberty, gonadotropin-releasing hormone (GnRH), hypothalamic-pituitary-gonadal axis (HPG), genomic imprinting, DLK1, MKRN3For, преждевременное половое созревание, гонадотропин-рилизинг-гормон (ГнРГ), гипоталамо-гипофизарно-гонадная ось (ГПГ), геномный импринтинг, DLK1, MKRN3

## Abstract

Precocious puberty (PP, OMIM 176400, 615346) is an autosomal dominant disorder caused by the premature reactivation of the hypothalamic-pituitary-gonadal axis. Genetic, epigenetic, and environmental factors play a decisive role in determining the timing of puberty. In recent years, genetic variants in the KISS1, KISS1R, MKRN3, and DLK1 genes have been identified as genetic causes of PP. The MKRN3 and DLK1 genes are imprinted, and therefore epigenetic modifications, such as DNA methylation, which alter the expression of these genes, can also contribute to the development of PP. The aim of this study is to determine the methylation index of the imprinting centers of the DLK1 and MKRN3 genes in girls with a clinical presentation of PP. The methylation index of the imprinting centers of the DLK1 and MKRN3 genes was analyzed in a group of 45 girls (age 7.2 ± 1.9 years) with a clinical presentation of PP and a normal karyotype using targeted massive parallel sequencing after sodium bisulfite treatment of DNA. The control group consisted of girls without PP (n = 15, age 7.9 ± 1.6 years). No significant age differences were observed between the groups (p > 0.8). Analysis of the methylation index of the imprinting centers of the DLK1 and MKRN3 genes revealed no significant differences between patients with PP and the control group. However, in the group of patients with isolated adrenarche, an increased methylation index of the imprinting center of the MKRN3 gene was observed (72 ± 7.84 vs 56.92 ± 9.44 %, p = 0.005). In the group of patients with central PP, 3.8 % of patients showed a decreased methylation index of the imprinting center of the DLK1 gene, and 11.5 % of probands had a decreased methylation index of the imprinting center of the MKRN3 gene. Thus, this study demonstrates that not only genetic variants but also alterations in the methylation index of the imprinting centers of the DLK1 and MKRN3 genes can contribute to the development of PP.

## Abstract (ru)

Преждевременное половое созревание (ППС, OMIM 176400, 615346) – заболевание, которое вызвано преждевременной реактивацией гипоталамо-гипофизарно-гонадной оси. В определении сроков полового созревания ведущую роль играют генетические, эпигенетические и экологические факторы. В последние годы варианты в генах KISS1, KISS1R, MKRN3 и DLK1 были идентифицированы как генетические причины ППС. Гены MKRN3 и DLK1 являются импринтированными, в связи с чем эпигенетические модификации, такие как метилирование ДНК, изменяющее экспрессию данных генов, также могут рассматриваться в качестве причины ППС. Цель настоящего исследования – определение индекса метилирования центров импринтинга генов DLK1 и MKRN3 у девочек с клинической картиной ППС. Анализ индекса метилирования центров импринтинга генов DLK1 и MKRN3 проводили в группе из 45 девочек (возраст 7.2 ± 1.9 года) с клинической картиной ППС и нормальным кариотипом методом таргетного массового параллельного секвенирования после обработки ДНК бисульфитом натрия. Контрольная группа состояла из девочек без ППС (n = 15, возраст 7.9 ± 1.6 года). Различий по возрасту между группами не выявлено (p > 0.8). Анализ индекса метилирования центров импринтинга генов DLK1 и MKRN3 не показал различий между пациентами с ППС и контрольной группой. Группа пациентов с изолированным адренархе имела повышенный индекс метилирования центра импринтинга гена MKRN3 (72 ± 7.84 против 56.92 ± 9.44 %, p = 0.005). В группе пациентов с центральным ППС 3.8 % пациентов имели пониженный индекс метилирования центра импринтинга гена DLK1 и 11.5 % – гена MKRN3. Таким образом, показано, что не только генетические варианты, но и нарушение индекса метилирования центров импринтинга генов DLK1 и MKRN3 могут быть причиной ППС.

## Introduction

Adolescence is one of the key stages of personality development, characterized by complex changes in the neuroendocrine system and other biological processes that lead to physical and sexual maturation. The appearance of secondary sexual characteristics before the age of 8 in girls and 9 in boys is defined as precocious puberty (PP), with an incidence of approximately 3.7 cases per 10,000 individuals. Recently, the prevalence of this condition has been increasing (Chebotareva et al., 2022; Alghamdi, 2023). According to the International Classification of Diseases (ICD-10), PP is categorized into E22.8 (conditions of pituitary hyperfunction, central precocious puberty) and E30.9 (unspecified disorder of puberty, including isolated thelarche and isolated adrenarche).

The timing of the onset of puberty is significantly influenced by the child’s gender, race, genetic predisposition, environmental factors, diet, and socioeconomic status (Sazhenova et al., 2023). For example, obesity and exogenous hormone intake can have adverse effects (Peterkova et al., 2021; Micangeli et al., 2023). However, over the past decade, several genes have been identified that are part of a complex network of inhibitory, activating, and regulatory neuroendocrine factors critical for controlling the onset of puberty. These include KISS1 (1q32.1) and its receptor KISS1R (GPR54, 19p13.3), as well as two imprinted genes that are normally expressed only from the paternal allele: DLK1 (14q32) and MKRN3 (15q11.2) (Roberts, Kaiser, 2020; Faienza et al., 2022). The primary mechanism of monoallelic expression of imprinted genes is allele-specific DNA methylation, which establishes differential methylation patterns on the two parental chromosomes.

DLK1 (OMIM 176290) encodes an EGF-like membranebound protein that belongs to the epidermal growth factor family, participates in the Notch signaling pathway, and regulates preadipocyte differentiation. It is expressed in neuroendocrine tissues, particularly in the adrenal cortex (Gomes et al., 2019; Macedo, Kaiser, 2019). The MKRN3 gene (OMIM 603856) belongs to the makorin family and plays a role in regulating the onset of puberty by inhibiting the release of GnRH from the hypothalamus, thereby delaying the oncet of puberty (Abreu et al., 2020). The MKRN3 gene encodes a protein containing a zinc finger RING domain, which is characteristic of most E3 ubiquitin ligases involved in intracellular protein degradation via the ubiquitin-proteasome pathway. The MKRN3 gene may interact with proteins associated with puberty, insulin signaling, RNA metabolism, and intercellular adhesion (Li C. et al., 2021).

The DLK1 and MKRN3 genes, like most imprinted genes, are regulated by imprinting centers. The DLK1 gene has two imprinting centers: the germline MEG3/DLK1:IG-DMR and the secondary MEG3:TSS-DMR, which is established after fertilization. MKRN3 is regulated by the germline imprinting center SNURF:TSS-DMR and is directly controlled by the somatic MKRN3:TSS-DMR. These imprinting centers are methylated exclusively on the paternal allele in somatic tissues, such as leukocytes and skin fibroblasts (Okae et al., 2014).

Imprinted genes are known to play a crucial role in the development of both the brain and the placenta, the organ responsible for nourishing the embryo. Disruptions in the hypothalamic-pituitary system, which regulates the endocrine activity of the brain during embryonic development, can adversely affect the formation of the fetal endocrine system (Tucci et al., 2019). The fetal pituitary gland produces hormones such as somatotropic, follicle-stimulating, luteinizing, and thyroid-stimulating hormones, which are essential for fetal growth and the regulation of puberty. Dysfunction in the production of these hormones can lead to intrauterine growth restriction or PP after birth (Canton et al., 2021). It is possible that some cases of PP are associated with disruptions in the imprinted state of the DLK1 and MKRN3 genes.

The aim of this study is to determine the methylation index of the imprinted regions of the DLK1 and MKRN3 gene imprinting centers in girls with a clinical presentation of PP

## Materials and methods

The molecular genetic analysis included 45 girls with PP and a normal karyotype, aged 7.2 ± 1.9 years. This group was divided into two subgroups: girls with hyperpituitary function (PP of central origin, ICD-10: E22.8, n = 26, age 7.6 ± 1.4 years) and those with unspecified PP (ICD-10: E30.1, n = 19, age 6.9 ± 0.8 years). The latter subgroup was further divided into girls with isolated thelarche (n = 11, age 7.4 ± 1.2 years) and isolated adrenarche (n = 8, age 6.8 ± 1.4 years). The control group consisted of girls without PP (n = 15, age 7.9 ± 1.6 years). No significant age differences were observed between the groups (p > 0.8). The patient cohort was recruited from the Scientific Center of Family Health and Human Reproduction Problems, Irkutsk. The study was conducted in accordance with the principles of the World Medical Association’s Helsinki Declaration. The study protocol was approved by the bioethics committee of the Scientific Center of Family Health and Human Reproduction Problems, Irkutsk (Protocol No. 1.1, dated January 12, 2023). Informed consent for participation in the study and DNA analysis was obtained from the parents or legal guardians of all participants.

Description of patient subgroups:

• Girls with the isosexual gonadotropin-dependent form of PP, under 8 years old, exhibiting accelerated physical development (height SDS +1 or more), with their sexual development corresponding to Tanner stages 2–4, levels of pituitary gonadotropic hormones corresponding to pubertal values, and a positive buserelin test. Additionally, they have enlarged mammary glands and uterus, as confirmed by ultrasound, and their biological age does not match their chronological (passport) age. • Girls with isolated enlargement of the mammary glands (thelarche), under 8 years old, with either accelerated or normal physical development (height SDS +1 or more), advanced sexual development corresponding to Tanner stage 2, levels of pituitary gonadotropic hormones corresponding to prepubertal values, and a negative buserelin test. Additionally, they exhibit enlarged mammary glands and uterus, which is confirmed by ultrasound. • Girls with isolated adrenarche, under 8 years old, with either accelerated or normal physical development (height SDS +1 or more), advanced sexual development corresponding to Tanner stage 2–3, levels of pituitary gonadotropic hormones corresponding to prepubertal values, and a negative buserelin test. Additionally, they exhibit enlarged mammary glands and uterus, which is confirmed by ultrasound.

All probands underwent standard cytogenetic analysis, which showed a normal karyotype in all cases. Karyotyping was performed using a research-grade microscope AxioImager (Carl Zeiss, Germany).

Genomic DNA was isolated from venous blood by phenolchloroform extraction. Bisulfite modification of DNA was performed using the EZ DNA Methylation-Direct Kit (Zymo Research, USA) according to the manufacturer’s protocol. During bisulfite conversion, unmethylated cytosine is modified to uracil, which is replaced by thymine during further PCR, and methylated cytosine is not modified. Methylation index analysis was performed using targeted bisulfite massive parallel sequencing.

To create libraries, specially designed oligonucleotide primers were used that allow amplification of target genome regions from bisulfite-converted DNA. Primers were selected for imprinted regions containing CpG dinucleotides of the MEG3/DLK1:IG-DMR and MKRN3:TSS-DMR imprinting centers, which control the expression of the DLK1 and MKRN3 genes, respectively. The UCSC genome browser (University of California, Santa Cruz), which contains information on genome sequences (GRCh38), was used to obtain the nucleotide sequence. The obtained nucleotide sequence was then used to select primers using the MethPrimer bioinformatics program (Li L.C., Dahiya, 2002). Vector NTI Advance 11.5 was used to test the thermodynamic properties of the primers.

The imprinted DLK1 gene is located on chromosome 14, at locus 14q32.2. Expression of this gene is regulated by the imprinting center MEG3/DLK1:IG-DMR, position 100,809,090– 100,811,721 (GRCh38). This genomic region contains 52 CpG dinucleotides, changes in the methylation index of which can affect the expression of this gene. Expression of the imprinted MKRN3 gene, located on chromosome 15, at locus 15q11.2, is regulated by the imprinting center MKRN3:TSS-DMR, position 23,561,939–23,567,348 (GRCh38). This genomic region contains 26 CpG dinucleotides, changes in the methylation index of which affect the expression of this gene (see the Table).

**Table 1.**
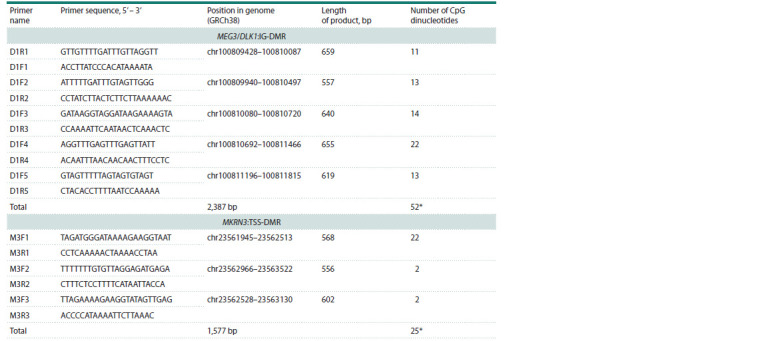
Sequences of the oligonucleotide primers used for analysis of the imprinting centers of the DLK1 and MKRN3 gene * Taking into account the overlap of the analyzed regions

Amplification of target fragments was carried out using the HS-Taq PCR kit (2×) (Biolabmix, Russia) according to the manufacturer’s protocol with the following PCR conditions: 95 °C for 5 min; 36 cycles: 95 °C for 20 s, 66 °C for 30 s, 72 °C for 40 s. The concentration of the target fragments was determined using a Qubit 4.0 fluorimeter (Thermo Fisher Scientific, USA). The reaction products were purified using Sephadex G50 solution (Sigma, USA). Targeted bisulfite massive parallel sequencing was performed on a MiSeq device (Illumina, USA) using a Micro kit (2x150).

The quality of the reads was evaluated using FastQC v0.11.8, after which the remaining adapter sequences and low-quality reads were trimmed using Trim-Galore. The reads were then mapped to bisulfite-converted target sequences using the bwa-meth tool (v0.2.2) with default parameters. Methylation data in the context of CpG were extracted from the resulting BAM files using the MethylDackel tool. The results were presented as the methylation index, which is the ratio of the number of cytosines to the total number of cytosines and thymines in a specific CpG site. In addition, the average methylation index was calculated along all target sites.

A limitation of the targeted bisulfite massive parallel sequencing used in this study is the impossibility of differentiating hypomethylation of the identified imprinting centers from uniparental disomy of chromosomes 14 and 15, as well as from microdeletions in these regions. Therefore, in case of detection of a decrease in the methylation index in the imprinting centers of the DLK1 and MKRN3 genes, real-time PCR was performed for the intergenic locus 14q32.3 and the NIPAI gene (15q11.2), respectively, to exclude deletion variants in these regions.

Statistical analysis was performed using the Statistica 10.0 software package (StatSoft, USA). The Mann–Whitney rank test was used to compare the methylation index between groups of samples. The differences were considered statistically significant at p < 0.05.

The study was conducted using the equipment of the center for collective use “Medical Genomics” of the Tomsk National Research Medical Center of the Russian Academy of Sciences.

## Results

**Fig. 1.**
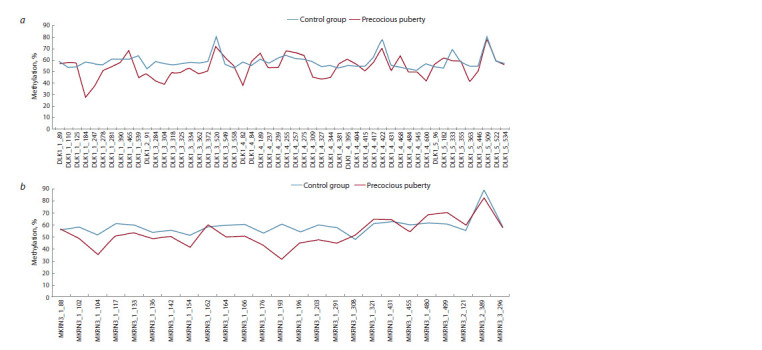
Methylation index of CpG dinucleotides of the imprinting centers of the DLK1 (a) and MKRN3 (b) genes.

Analysis of the methylation index of 52 CpG dinucleotides of the imprinting center of the DLK1 gene (MEG3/ DLK1:IG-DMR) in the control group showed that three loci (DLK1_3_520, DLK1_4_422, and DLK1_5_509) are hypermethylated, which is atypical for imprinted genes (Fig. 1a). When superimposing the methylation index of the control group and the group of probands with PP for these CpG dinucleotides, it is also evident that these loci have an increased methylation index and do not differ from the control group (Fig. 1a). Therefore, these loci were not included in the present study. In contrast to the DLK1 imprinting center, only one of the 25 CpG dinucleotides (MKRN3_3_296) of the MKRN3 gene imprinting center (MKRN3:TSS-DMR) in the control group was atypically hypermethylated (Fig. 1b). When comparing the methylation of the control group and probands with PP, an increased methylation index was also observed for this locus (Fig. 1b). Therefore, this locus was also not included in further analysis

**Fig. 2.**
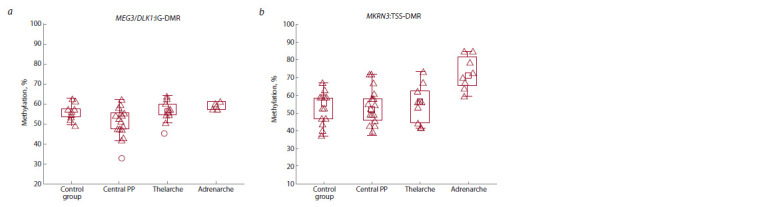
Pairwise comparison of the mean methylation index of the DLK1 and MKRN3 imprinted genes between the groups.

The average methylation index of the DLK1 imprinting center in probands with PP was not significantly different from the control group (51.46 ± 9.59 % vs 58.44 ± 7.15 %, respectively, p = 0.058). Although a slight decrease in this index was noted, it was not statistically significant (Fig. 1a). Girls with PP were then divided into three subgroups based on their symptoms: central PP, isolated thelarche, and isolated adrenarche. Pairwise comparisons between these subgroups also failed to reveal significant differences in the methylation level of this gene (Fig. 2a): 58.44 ± 7.15 % in the control group, 53.32 ± 8.56 % in the group with central PP, 57.40 ± 9.31 %, with isolated thelarche, and 59.74 ± 2.05 %, with isolated adrenarche (p > 0.05). Comparison of the methylation index of each patient with PP and the control group revealed that only one patient with central PP showed a significant decrease in methylation (31.16 % vs 58.44 ± 7.15 %, p < 0.01). Consequently, in the PP group, there is a decrease in methylation levels in the imprinting control region of the DLK1 gene in 2.22 % (1/45) of patients, and in girls with central PP, this decrease occurs in 3.84 % (1/26) of cases.

Analysis of the methylation index of the imprinting center of the MKRN3 gene showed no significant differences between patients with PP and the control group (53.71 ± 10.30 % vs 56.92 ± 9.26 %, p = 0.71). However, when the group with PP was divided into three subgroups (central PP, isolated thelarche, and isolated adrenarche), there was a significant increase in the methylation index in the adrenarche group compared to the control (72.00 ± 7.84 % vs 56.92 ± 9.44 %, p < 0.005) (Fig. 2b). Comparison of the methylation level of each patient with the control group revealed that three patients with central PP had reduced levels (34.09, 29.01, and 32.08 %, compared to 56.92 ± 9.44 % in the control, p < 0.01). Therefore, 6.66 % of patients (3/45) in the PP group and 11.53 % of patients in the central PP group (3/26) had reduced methylation levels of the MKRN3 imprinting center.

The decrease in the methylation index of the imprinting centers of the DLK1 and MKRN3 genes may indicate both uniparental disomy of chromosomes 14 and 15 of maternal origin and deletions of these regions on chromosomes of paternal origin. Deletion variants that included the imprinting centers of the DLK1 and MKRN3 genes were excluded by realtime PCR. Uniparental disomy for chromosomes 14 and 15 was not excluded. At the same time, uniparental disomy of chromosomes 14 and 15 is a very rare event (1:50,000 people for chromosome 15) (Butler, 2020).

Thus, in the present study, the analysis of the methylation index of the imprinting centers of the DLK1 and MKRN3 genes between patients with PP and the control group showed no significant differences between the two groups. However, the group of patients with isolated adrenarche had a significantly increased methylation index of the MKRN3 imprinting center (72.00 ± 7.84 % vs 56.92 ± 9.44 %, p < 0.005). In the group of patients with central PP, 3.8 % of patients had a decreased methylation index of the DLK1 gene, and 11.5 %, that of the MKRN3 gene. The presence of epimutations simultaneously in two loci was not detected in any proband.

## Discussion

In recent years, there has been an increasing understanding of how epigenetic factors influence the timing of puberty. The current study found a statistically significant reduction in the methylation level of the imprinting centers for the DLK1 and MKRN3 genes among a group of girls with central PP. This reduction was observed in 3.84 % of patients for DLK1, and 11.53 %, for MKRN3. It is worth noting that a decrease in methylation level (hypomethylation) at the DLK1 imprinting site has also been reported in 8.8 % of individuals with Temple syndrome (Narusawa et al., 2024), a disorder characterized by pre- and postnatal growth retardation, hypotonia, feeding difficulties, delayed motor development, joint laxity, trunk obesity, and facial dysmorphisms (e. g., broad forehead, short nose, micrognathia). In some cases, an early onset of puberty has also been reported (Kagami et al., 2019).

The core clinical features of Temple syndrome are similar to those of Russell–Silver (OMIM 180860) and Prader–Willi (OMIM 176270) syndromes (Kagami et al., 2015; Abi Habib et al., 2019). In the present study, a decrease in the methylation index of the DLK1 gene imprinting center was found in one proband with PP, who also had obesity, pericallosal lipoma, and hypogenesis of the corpus callosum. In addition to PP and obesity, the clinical picture of the patient did not correspond to the phenotype of Temple syndrome. According to the literature, Canton et al. (2020) showed a decrease in the methylation index of the DLK1 imprinting center in 2.5 % (3/120) of patients with PP, prenatal and postnatal growth retardation, hypotonia, and feeding difficulties. Further study of these cases revealed the presence of uniparental disomy of chromosome 14 of maternal origin in two of them

Hypomethylation of the imprinting center of the MKRN3 gene may be associated with dysfunction of other genes that are normally imprinted and expressed only from the paternal chromosome. These genes include MAGEL2, NECDIN, NPAP1/C15orf2, SNURF-SNRPN, MKRN3-AS/ZNF127- AS, UBE3A-AS, IPW, SNORD116, SNORD115, SNORD64, SNORD107, SNORD108, SNORD109A, and SNORD109B.

Uniparental disomy of the maternal chromosome 15 or deletion of the paternal copy of these genes can cause Prader–Willi syndrome. The clinical picture of this syndrome includes muscular hypotonia, obesity, hyperphagia, short stature, hypogonadism, and mental retardation of varying severity. Rarely, this syndrome can be accompanied by PP (Nicoara et al., 2023). Patients with hypomethylation of the imprinting center of the MKRN3 gene, as identified in this study, had central PP, but without the clinical features of Prader–Willi syndrome. Deletion of the 15q11-q13 region was excluded in these patients. If they had uniparental disomy of this region from the mother, it would certainly lead to the clinical picture of Prader–Willi syndrome. Therefore, it seems that these patients likely only had hypomethylation of the MKRN3 imprinting center.

An increase in the methylation index of the imprinting center of the MKRN3 gene has also been shown in the group of probands with isolated adrenarche. Premature adrenarche in children is not caused by premature activation of the hypothalamic- pituitary-gonadal axis, but it is associated with excessive activation of the reticular zone of the adrenal glands, which is the source of dehydroepiandrosterone (DHEA). DHEA is converted into testosterone and dihydrotestosterone in peripheral tissues. During intrauterine development, the adrenal glands start to produce DHEA, which is used to produce placental estriol. A timely increase in androgen levels is necessary for puberty, promoting growth and strengthening of bone tissue, as well as stimulating the process of red blood cell production. Moderate levels of DHEA activate the development of the prefrontal cortex in the brain, providing neuroprotective and neurotrophic effects, and regulate the function of GABA receptors, which are the main inhibitory neurotransmitters in the central nervous system.

Hypermethylation of the MKRN3 imprinting center leads to increased expression of the gene, resulting in an increased amount of protein produced. MKRN3 then ubiquitinates various factors to suppress the production of gonadotropin-releasing hormone 1 (GNRH1). Ubiquitination of the transcriptional repressor MBD3 inhibits its binding to both the GNRH1 promoter and DNA TET2 demethylase, resulting in epigenetic silencing of GNRH1 transcription. Additionally, MKRN3 mediates the ubiquitination of poly(A)-binding proteins, such as PABPC1, PABPC3, and PABPC4. This reduces their binding to the poly(A) tail of target mRNA, including GNRH1 mRNA. This affects the stability and translation of these mRNAs (Li C. et al., 2020; Fanis et al., 2022). The MKRN3 expression is also found in the adrenal glands, although its role in activating adrenarche remains unknown.

Genome-wide, exome-wide, and methylome-wide association studies on the pathogenetic heterogeneity of PP have shown that genetic variants in the KISS1R, KISS1, MKRN3, and DLK1 genes occur in approximately 10 % of sporadic cases and 27 % of familial cases. Copy number variations (7q11.23 deletions, Xp22.33 deletions, 1p31.3 duplications) are found in 4 % of patients. Epigenetic abnormalities at the imprinting centers of the DLK1 and MKRN3 genes are rare, as reported by Canton et al. (2020). Whole exome sequencing has also identified rare, de novo variants that cause loss of gene function in the dominant state. These include the TNRC6B (22q13.1), AREL1 (14q24.3), PROKR2 (20p12.3), and LIN28B (6q16.3) genes, although their roles in the disease process are not fully understood (Shim et al., 2022).

Methylome analysis of this pathology has shown the presence of hypomethylation in the promoter region of the ZFP57 gene (6p22.1) (Bessa et al., 2018). ZFP57 contains a KRAB domain, which is a transcriptional repressor, and different genetic variants of this gene have been linked to multi-locus imprinting disturbance (MLID), such as transient neonatal diabetes mellitus (OMIM 601410). This condition is accompanied by hypomethylation of several other imprinted genes, including PLAGL1, GRB10, and PEG3 (Monteagudo-Sánchez et al., 2020; Mackay et al., 2024). The expression of the ZFP57 gene in the hypothalamus of female rhesus macaques has been shown to increase during peripubertal development. This indicates an enhanced repression of downstream target genes of ZFP57 (Bessa et al., 2018). The increased expression of ZFP57 has been observed in the hypothalamus of mature female monkeys. This indicates that this gene may play a role in suppressing the activity of transcription repressors involved in puberty, such as the Polycomb complex (Lomniczi et al., 2015).

Thus, it has been shown that not only genetic variations, but also a disruption of the methylation pattern of the imprinting regions of the DLK1 and MKRN3 genes, can be a cause of PP.

## Conclusion

Puberty is a multifactorial process with a triggering role of genetic and epigenetic factors. In this study, no significant changes in methylation of the imprinting centers of the DLK1 and MKRN3 genes were shown between patients with the clinical picture of PP compared to the control group. Despite this, it was found that a group of patients with isolated adrenarche had an increased methylation index of the imprinting center of the MKRN3 gene. Among the girls with central PP, 3.8 % had a decreased methylation index for the imprinting centers of the DLK1 and 11.5 % of the MKRN3 genes, and the total contribution from methylation disorders in these genes was 15.3 %. No probands had epimutations in both of these loci at the same time.

Therefore, it was demonstrated that not only genetic, but also epigenetic changes may be responsible for PP through methylation disorders in the imprinting regions of the DLK1 and MKRN3 genes. However, there are some limitations to the conclusions drawn, and there may be other epigenetic factors that can also influence the formation of PP, such as epimutations in other imprinted genes or mutations in the imprinting centers that control the expression of imprinted genes. Nevertheless, this study shows the epigenetic role of the imprinted genes DLK1 and MKRN3 in the development of PP

## Conflict of interest

The authors declare no conflict of interest.
